# Barriers and facilitators to implementation of interventions to mitigate moral injury among nurses

**DOI:** 10.3389/frhs.2025.1582700

**Published:** 2025-06-23

**Authors:** Cassandra M. Godzik, Jennifer K. DiBenedetto, Timothy J. Usset, Heather Stiles, Heather Klein, Karen Fortuna, Renee Pepin, Hannah Wright, Amy Locke, Helen Thomason, Andrew J. Smith

**Affiliations:** ^1^ Department of Psychiatry, Dartmouth-Hitchcock Health System, Lebanon, NH, United States; ^2^ Geisel School of Medicine, Dartmouth College, Hanover, NH, United States; ^3^ Department of Mental Health, VA Maine Healthcare System, Augusta, ME, United States; ^4^ School of Public Health, University of Minnesota, Minneapolis, MN, United States; ^5^ Department of Psychiatry, University of Utah, Salt Lake City, UT, United States; ^6^ Department of Family & Preventive Medicine, University of Utah, Salt Lake City, UT, United States

**Keywords:** moral injury, nurses, medical intensive care unit, intervention development, intervention implementation

## Abstract

**Background:**

In the post-pandemic recovery era, addressing moral injury is critical due to high prevalence and impact on mental and occupational health. Interventions that address moral injury in hospital settings are limited. Further, engaging HCWs in any mental health interventions has proven challenging for a variety of reasons and exacerbated by factors such as a rural setting. Implementation science aimed at understanding barriers and facilitators to interventions is needed in order to build and offer interventions that are usable, feasible, acceptable, and effective. The current study aimed to understand such barriers and facilitators to building moral injury interventions for nurses on the medical intensive care unit (MICU).

**Methods:**

We conducted semi-structured qualitative interviews using the Consolidated Framework for Implementation Science Research (CFIR) and Peer and Academic Model of Community Engagement with 25 participants in a rural hospital system, 19 nurses currently working in the MICU and six nurses who left their MICU employment. Interviews were transcribed and analyzed using a thematic analysis approach.

**Results:**

There were five CFIR domains and 14 associated CFIR constructs that impacted intervention implementation in this population. Barriers included resource costs, skepticism regarding the effectiveness of new resources, lack of support from leaders, concerns that emotions affect professional image, inability to take breaks, and a disconnect between nurses' lived experiences and community perceptions. Facilitators included interventions specifically tailored for the MICU, strengths in teaming and social support among fellow nurses, and a desire for change because of factors such as a high turnover rate. Participants also highlighted a strong motivation to provide the best care possible and a desire to build resilience by supporting each other.

**Conclusion:**

Analysis of barriers and facilitators suggests value in improving the opportunities for HCWs to process morally injurious experiences with interventions specific to a particular unit and resources such as peer support and chaplains. There is a demonstrated need for high-level organizational change to address the dynamic needs of our nurses.

## Introduction

The COVID-19 pandemic exacerbated long-standing distress among healthcare workers (HCWs) (1–7). Downstream effects of this distress manifest in systemic short staffing that is not projected to be easily solved without intensive effort and intervention (8). Recent longitudinal findings hone-in on exposure to potentially morally injurious events (PMIEs) as a key driver of burnout and turnover intentions among HCWs (9). This longitudinal evidence supports the recent explosion in editorial and empirical work from frontline HCWs, leaders, and scholars to build understanding and interventions specifically to mitigate the harms from rampant moral injury in healthcare (10). Aligned with this evidence, this problem space, and these calls for innovation, the current study utilized an implementation science approach to uncover barriers and facilitators to developing and implementing interventions to mitigate the harms driven by moral injury.

Moral injury is defined as the consequences of experiencing, participating-in, or witnessing moral transgressions that violate deeply held beliefs. It can also include experiences of betrayal from leaders or authority figures in high stakes situations (11, 12). Evidence shows that ∼75% of HCWs may be experiencing exposure to potentially morally injurious events (PMIEs) within their occupational environments (13, 14). Among HCWs exposed to PMIES, approximately 50% experience downstream functional impairments that are consistent with moral injury presentations (10), including impaired interpersonal relationships and onset of mental health disorders (15, 16). Moral injury in nursing stems from the experience of transgressed beliefs and values from a variety of sources, including patient violence events, unit disunity, lack of resources to provide evidence-based practice care, conflicts of interest between financial and clinical priorities, and leadership failures (12, 17, 18). These experiences can have severe occupational consequences, particularly in healthcare settings struggling to maintain quality care due to staffing shortages (19). In fact, as of December 2020, more than one in three nurses expressed intentions to leave their organizations or occupations (20). In 2024, the average RN vacancy rate was found to be 9.6% with over 40% of institutions reporting vacancies of 10% or more (21). This desire for nurses to leave the profession is a reality for many hospital roles, where general hospital turnover rates have reached as high as 25.9% in 2021 (21). While turnover rates have improved by 7.6% since 2021, it is unlikely that retention will improve unless we address moral injury in the healthcare system (9, 20, 21).

There is a plethora of evidence-based interventions and strategies to address moral injury in military populations (22, 23). However, it is unclear how we would effectively engage the nursing workforce in these interventions. Our ability to develop and engage nurses in interventions is very limited. Most HCWs endorse that they would seek help if in hypothetical emotional distress, only a small percentage do seek help when in actual emotional distress (24, 25). Nurses in need suffer from low treatment uptake, highlighting the likelihood that there are barriers to care that require elucidation in order to move towards innovation (26, 27).

There is a nascent evidence base for understanding barriers and facilitators to nurse uptake of interventions. It can be difficult to access mental health services due to schedule (nights, weekends) and shift work. HCWs, including nurses, may worry that identifying a psychiatric condition or engaging in mental health services could impact licensure or employment status (28). Short staffing, which has become endemic in healthcare, compounds these challenges by limiting nurses' time and ability to engage in additional activities either during or outside of routine work (29). The culture of nursing can be unproductive for help seeking as well, fundamentally valuing self-sufficiency, discouraging vulnerability, and rewarding overwork (30). In rural healthcare settings, nurses face access problems given the dearth specialized mental health professionals and available interventions (31). As a result of these barriers, many nurses attempt to manage mental health and occupational challenges through a variety of naturalistic strategies that vary from adaptive to maladaptive (32), ranging from things like seeking social support to medication and alcohol misuse (6, 33, 34). The MICU nursing population is particularly vulnerable because they experience PMIEs at a high rate since their patients have high rates of mortality and morbidity (35).

The current study employed the 2022 Consolidated Framework for Implementation Science Research (CFIR) to improve our ability to build usable, effective interventions that can be widely disseminated to nurses. CFIR is a well-established tool for researchers, usually in the healthcare field, to assess and improve multiple factors that can influence the success of implementation efforts (36). Our focus was on engagement through this methodology (which constitutes the focus of the current paper), as part of a larger goal and overarching research program to develop and implement a feasible, acceptable, appropriate, and efficacious proof of concept intervention that nurses will utilize to mitigate the harms caused by moral injury (37). This discovery process involved exploration of nurse beliefs and values, moral injury experiences, barriers and facilitators for coping, processing and support, and perspective on intervention components and mediums that would facilitate effectiveness and engagement.

## Methods

### Recruitment

Two MICU nurses, who were both part of this research team and embedded (currently employed) in the MICU, approached other MICU nurses and shared at monthly staff meetings (single MICU in a rural healthcare system) the current research study, its purpose, and provided research team contact information. Nurses who no longer worked at this MICU were also contacted by the embedded MICU nurses who had worked with them previously; contact was initiated by email. Interested participants contacted the research team; all data collection occurred either on-site, in-person at the MICU in a private room, or via Zoom. Interview duration lasted from approximately 20–60 min. Participants were not provided any form of compensation for their time.

### Participants and setting

The criteria for RN eligibility to participate in the study included (1) current employment in the MICU for at least 3 months or (2) recent employment in the MICU. They were recruited from a single MICU in a rural academic healthcare system located in the Northeast U.S.

### Interview guide

The interview guide was developed based on the Consolidated Framework for Implementation Science Research. CFIR is a conceptual framework that was developed to guide the systematic assessment of multilevel implementation contexts to identify factors that might influence intervention implementation, including barriers and facilitators that contribute to its effectiveness (36). The five domains of the established CFIR framework include the following: (I) intervention characteristics, (II) characteristics of individuals, (III) implementation process, (IV) inner setting, and (V) outer setting. The interview guide included a definition of moral injury and five open-ended questions/probes as follows: (1) What are your beliefs, values, or moral principles that guide your work in healthcare?; (2) Do you think these types of problems are pertinent or applicable in your work on the MICU?; (3) If you have personally experienced a potentially morally injurious event, tell me about a time that you did; (4) Tell me about your experience since the event (or events) that you just described. What have you done to cope? Is the event (or events) continuing to impact you, and how?; and (5) Let's talk about the people who have been there for you, and how you felt supported by them.

The semi-structured interview guide was co-produced with current MICU nurses (not interviewed for this study but part of the current research team—HS, HK), other research team members with expertise in Moral Injury (AM, AS, TU, CG, KF), and collaborators at another academic medical center (HW, MC, MV) using the Peer and Academic Model of Community Engagement (38). Interviews were audio recorded via secure Zoom platform and transcribed by two research team members (HS, HK) and checked for accuracy by two additional members (CG, JD). All identifying information was deleted from the transcripts, and the audio recordings were destroyed. Interviews were conducted until data saturation (i.e., saturation means that sampling more data will not lead to more information related to research questions) (39). Member checking, verifying the results with the employed and unretained nurses, was employed. It is a technique for exploring the credibility of results (generated by the research team) where the data or results are returned to participants to check for accuracy and resonance with their experiences (40).

### Ethical considerations

This study involved semi-structured qualitative interviews with nurses currently employed in the MICU (retained) and nurses who recently left the MICU (unretained). This research was part of a quality improvement project to improve nurses' experience and develop opportunities to improve nursing well-being in the MICU setting. It was reviewed by the Dartmouth Institutional Review Board (IRB).

### Data analysis

The data analysis was informed using a thematic analysis approach (i.e., a method for identifying, studying, and communicating patterns in the data material) (41). Research team members, including nurse scientists (CG, JD), a chaplain scientist (TU), embedded MICU nurses (HS, HK), and community-partnered researchers (RP), read all the interview transcripts independently to familiarize themselves with the data. These authors assigned data-driven codes to text segments representing relevant data findings aligned with the research purpose. The codes were collated and grouped into preliminary themes documenting reoccurring concepts or statements reported by the research subjects. Research team members met virtually for five sessions and discussed the assigned codes, their relations to the themes, and characteristics of the naming of each theme. Five research members were involved in the coding process for validation purposes and broadening the analyses' breadth and depth (42). Data were triangulated to examine similarities and differences in responses. Research members agreed on the naming of the themes and placement of themes under the CFIR domains.

## Results

### Participant demographics

This study involved 25 participants (*N* = 25), including retained nurses, those currently working in the MICU (*N* = 19), and unretained nurses, those who have left their employment in MICU (*N* = 6). Nurses had a mean age of 33.5 years and were primarily women (59.5%), white (84%) with 7.2 average years of nursing experience (Table 1).

**Table 1:** Demographics of study participants (*N* = 25).

Employment status (Retained/Unretained)	Age range (years)	Sex (percentages)	Race (percentages)	Time in nursing career range (years)	Hours worked per week (hours)
Retained = 19; Unretained = 6	24–62	59.5% females; 40.5% males	White (84%), black (5%); Asian (5%); not disclosed (6%)	0.5–41	54.11 (38.8)

### CFIR themes

Overall, there were eight barriers and six facilitators identified to be important in the design of a moral injury intervention for MICU nurses (Table 2). Barriers identified included: (I) intervention characteristics —innovation relative advantage and innovation cost, (II) characteristics of individuals—high-level leaders, (III) implementation process—engaging, (IV) inner setting—implementation readiness, work infrastructure and available resources, and (V) outer setting—local conditions. The facilitators included: (I) intervention characteristics—innovation adaptability, (II) characteristics of individuals—motivation and need, (III) implementation process—teaming and adapting, and (IV) inner setting—tension for change. These findings are described by themes corresponding with their CFIR domain, respectively.

**Table 2:** CFIR domains and constructs.

CFIR domains	CFIR constructs	
	Barriers	Facilitators
Overall intervention characteristics- *Barriers and facilitators to accepting intervention*	1. Innovation cost 2. Innovation relative advantage	1. Innovation adaptability
Characteristics of individuals- *Barriers and facilitators to intervention implementation*	3. Lack of support and engagement from high-level leaders	2. Motivation of nurses 3. Nurse need to interventions to help with morally injurious events
Implementation process- *Barriers and facilitators to intervention implementation*	4. Engaging (implementation characteristic)	4. Teaming (relying on team members) 5. Adapting (want to participate in activities that promote group growth)
Inner setting- *Barriers and facilitators of potential intervention location*	5. Existing resources (implementation readiness) 6. Work infrastructure 7. Availability of resources	6. Tension for change
Outer setting- *Barriers and facilitators to intervention effectiveness*	8. Location conditions	

### Theme I: overall intervention characteristics

#### Barriers and facilitators to accepting intervention

The intervention characteristic, innovation cost, was reported to be a disadvantage or barrier to implementing moral injury interventions in the MICU. A specific number of sessions (mental health) are permitted to nurses and accessing these services through the institution has a long waitlist.

“It discouraged a lot of nurses from seeking those resources because there was a limit to it”.

Another barrier is innovation relative advantage, in that nurses do not feel that the options to support moral injury is any better than what already exists and is available to them. For example, one nurse described how there is infrequent follow-up to ensure that the nurses in distress are getting the care they need.

“There is no follow-through. Yes, you can get your 3 appointments. Yes, you can see a consultant psychiatrist once, but then that's it … if they had more continuity of care or a follow-up or help to direct you to the right resources and obtain them prior to letting go of you, [that] would be nice”.

Nurses' views on how an intervention can be adapted, or innovation adaptability, to meet their needs was a facilitator to intervention implementation.

“I think it has to be tailored to the traumatic experiences that are experienced here that are not commonplace elsewhere”.

### Theme II: characteristics of individuals

#### Barriers and facilitators to intervention implementation

There was a reported lack of support and engagement from high-level leaders that presents as a barrier to potential moral injury interventions. Examples describe times where management, who are supposed to be supportive leaders, show superficial support, but do not follow-through with their public messaging.

“They came out once [management] … and we really didn't see them again,” and “when the executive leadership comes on to the unit accompanied with a camera crew and they're asking you, how are you doing it? You're heroes! And then they don't come back. It's less than authentic”.

Nurses described the ever-changing and fast-paced work environment in this setting. This capacity for resilience was necessary to care for each other (other nurses) and family members as death is common in the MICU. The motivation of nurses is a facilitator to interventional work so they can build their resilience together.

“At the end of the day, the best support system were people that were right there with you. You could take your PPE off, go to the nursing station, and just vent for a minute … a lot of us became very close, tight-knit cause we only had each other”.

Another facilitator was the need for interventions to help with morally injurious events. Many nurses described moral injurious events such as, “My belief is always going and making sure that my patients get the equal amount of care that they deserve. No one is getting less of care. There were times in Covid where you couldn't really give all the care that you possibly can in a 12-hour shift just simply because of all the PPE, all the alarms going off … I watched those people suffer when there wasn't really as much we could do”.

### Theme III: implementation process

#### Barriers and facilitators to intervention implementation

The implementation characteristic, engaging, was reported to be a disadvantage or barrier to the implementation of moral injury interventions in the MICU. Some felt that sharing emotions and feelings could be seen as “weak” and their vulnerability to lead them to be less effective in care they provide as nurses on the unit.

“People don't want to become known as the complainer. There is a lot of shame put on people for speaking up or having emotion. I think there's a culture of being tough that is going to be really hard to change. People are really not wanting to seem vulnerable and that makes things tricky. I think it would have to be anonymous”.

There were, however, facilitators that were described by participants including teaming where they felt they were part of a team they could rely on during times of difficulty they face on the unit, and it can be difficult to allocate time to do so during the busy work shifts.

“I think all of us bedside nurses really kind of leaned on each other. It was really nice …. there were a lot of nurses [and] we kind of got together, and it was nice to have everyone to lean on. And so that was good to have a group of people that you can kind of share in this trauma with”.

Another facilitator, adapting, was seen as a theme during the interviews when participants talked about how they want to participate in interventions that help their team to grow; however, they do not know how to make it work. One participant stated, “I think that what we're doing now doesn't work as evidenced by the ridiculously high turnover rate in nursing in general. The status quo has failed and will continue to fail. So, I think any innovation or thoughts, I am here for”.

Another nurse noted the importance of in the moment chaplain support when caring for a very sick patient: “We had a really active Chaplain on the floor whenever there was like a really sick individual, the charge nurse for that day was always very present, and checking in both with like, the nurse and like the like pod partner that was down there. The camaraderie and like staff were a little closer knit”.

### Theme IV: inner setting

#### Barriers and facilitators of potential intervention location

Nurses reported existing resources, or implementation readiness, were not aligned with the needs of nurses related to their experience of morally injurious events on the MICU and is seen as a barrier. There is a lack of available and trained mental health treatment services. EAP was experienced in negative ways, where on the unit chaplain support was experienced more positively.

“I went to EAP in the beginning of Covid, and they weren't helpful. They didn't know how to deal with the trauma, and you only get 6 free sessions. 6 free sessions, for a nurse going through Covid, is not enough. I feel like having them more accessible on rounding on the unit as well as like our chaplain, that runs on the unit and checks on us personally as well as our patients”.

Work infrastructure was also seen as a barrier to development of interventions on their units. Time to set aside to process a difficult situation, where it is not only a debrief of what happened, but a place to share the emotional experience of what occurred is not possible, currently.

“I need the support from my co-workers and my leadership and our staffing to be able to accommodate that, so that I can, after dealing with something distressing, take half an hour to come in, sit, detach from my other patient and actually talk about what happened”.

The third barrier was available resources where there are things offered such as float staff and mental health sessions, but it does not feel that there are enough to go around for all nurses to use. An example from a participant is:

“And then even when you get those resources, part of you is like, you're taking it away from another unit who could also benefit from it”.

There was a facilitator, tension for change, described by participants where the current situation is untenable and is not working. Some report taking the necessary steps to make changes in their personal lives. One person reports, “And so balancing that with, like, you know, young kids at home, and I just felt like my mental health took a huge dip during that time, and I there was no way that I thought to back down, and you know, stay in the MICU … and so I did a 180 and I left”.

### Theme V: outer setting

#### Barriers and facilitators to intervention effectiveness

Local conditions were seen as a barrier where there was a disconnect between what they experienced as nurses and how the community perceived and celebrated their work, as nurses. This was highlighted in multiple participants on how they were celebrated with incentives including free meals from the community when they were continuing to face difficult cases in the unit, and the “rest of the world just moved on” after COVID.

“It happened at the one-year anniversary where I feel like it all hit me. That whole year, I was working so hard, and I felt like I was covering things up and burying these feelings. I didn't think I had a problem. I didn't think I was upset because I was working so hard to be excellent. Then, when they said it's our one-year anniversary and tried to celebrate that, it really hit me hard. That's when I started feeling emotional about this situation and I was like, how can you call this an anniversary when we just went through so much hardship and hurt and mental strain taking care of these people?”

## Discussion

This research study aimed to understand the barriers and facilitators MICU nurses face to addressing moral injury. The barriers and facilitators identified during this study can inform the development and implementation of moral injury interventions that may be relevant, feasible, and consider nurses' unique position (37). Interview data highlighted that morally injurious events were experienced by nurses at multiple levels across the healthcare system (i.e., with patients, colleagues including other nurses, and leadership). Moral injury is a multifaceted experience for MICU RNs because PMIEs involve various stakeholders including patients, families, peers, and administration/leadership. Based on facilitators that nurses described, addressing moral injury at multiple levels of the MICU by using a multilevel intervention approach may promote positive mental wellbeing among MICU nurses. Chaplains were specifically named as a positive source of support by MICU nurses in contrast to the more neutral, or negative statement about formal mental health support. This may be associated with the unique role that healthcare chaplains have for directly caring for both patients and staff—time spent on units with frontline HCWs—that may have helped them be more approachable and trusted by the MICU nurses because they worked together on a day-to-day basis.

Theme II, characteristics of individuals, noted experiences with discordance between nurses and their management and how it affected implementation. Although it may be common knowledge for frontline nurses to view patient care as a priority, leaders may hold a different belief system on how that care should be delivered (32, 43, 44). Charge nurses (who oversee frontline/bedside nurses) have worked as bedside nurses prior to promotion to “charge” nurse (45). Conversely, executive nurse leaders in the organization may either not hold that same level of bedside experience (direct care) or be further removed from their time as bedside care providers; thus, taking direction from executive nurse leaders may not be consistent with the moral belief system held by charge nurses or floor nurses (46). The difference in attunement to the unit's needs extends to variable phenomenology of the experiences of moral distress at different “levels” in the chain of command. For example, if one were to say that moral injury, perhaps, erodes compassion by making every member of the workforce a victim to it; then to live in a victim stance promotes the justification of perpetrating moral injuries to the next patient or HCW. Unless we have knowledge of how moral injury is experienced across the hierarchy \nursing (e.g., floor nurse, charge nurse, unit nurse manager, department leadership, executive management, etc.), an opportunity can be missed to inform compassion-oriented interventions that allow nurses to work together and with the support of their leaders to combat moral injury.

Theme I, innovation characteristics, included the barrier “innovation relative advantage”. Many of the potentially available interventions described did not benefit nurses more than their current natural coping mechanisms. Reading the data suggests that for interventions to offer relative advantages in currently high moral distress environments, the characteristics of interventions must initiate validation of the work that nurses do that exposes them to PMIEs, upon which shared experience and narratives can be solidified. From a foundation of validated PMIE exposure, we can adapt existing interventions to empower individuals to act towards positive social meaning, service, and connection despite complex parameters (e.g., inevitable PMIEs, difficult leaders and/or patients or families; personal distress). The relative advantage offered would include this personal growth.

These specific findings underpin the need to approach intervention design from group and systems level approaches that can capitalize on shared experience and connection among nurses, which are vastly underdeveloped at current time. Currently, the vast majority of potentially available interventions for HCWs address problems at the individual/personal level of responsibility, mental health, and healing (8, 47), despite the fact that the majority of HCWs lack interest in such individual level approaches (8, 48, 49), and a very low percentage of nurses in distress actually use these types of interventions (49). Further, as individuals, HCWs including nurses are some of the most resilient people in our populus (30). The need for group and system approaches is further reinforced by the literature showing most PMIEs in healthcare are in the betrayal and witnessing domains. Although some HCWs will certainly benefit from moral injury interventions at the individual level, continuing to prioritize approaches to moral injury (and HCW well-being in general) as individual phenomena (e.g., as a mental health or resilience deficit) is likely to be experienced as invalidating and in its own way a perpetuation of further PMIEs. Individual interventions are not mechanistically designed to treat structural problems in healthcare and unwittingly push the onus for care onto the nurse or physician instead of the system.

Beyond content of interventions, the “delivery” mechanism must attend to scarce resources, enormous problem scope, and the social etiology of moral injury. Innovation cost and innovation relative advantage were two sub-themes of intervention characteristics that identified the importance of affordability and availability in interventions. It is well established that effective delivery must aim to empower grassroots, unit-level networks, and ease of access to stimulate cultural healing from within (rather than from a top-down approach) (50). The interviews echo this sentiment and suggest the importance of intervention delivery at the level of nursing teams/units. They also suggest delivery of resources by peers or chaplains. Rather than continued pushes for more of the same mental health resources, the resources need to change. There is an urgent need for organizational change within healthcare institutions to properly address the needs of nurses facing PMIEs.

Theme III, implementation process, includes “teaming” as a facilitator. Debriefing in a group with other nurses with a lived experience of a moral injury, facilitated by a peer (a nurse with a lived experience of addressing moral injury) may offer a safe and structured way for nurses to process emotions and develop moral injury-coping strategies. Peer support within the mental health system has decades of history in improving mental health symptoms in people. Recent evidence shows that when HCWs engage in social support among peers, they improve their ability to cope with stressful and traumatic exposures and reduce risk for longer term consequences (51). Peer support has expanded to multiple groups outside of patients in community mental health centers and is now supporting physicians, social workers, and a natural extension may be nurses.

## Future directions

The next phase in this ongoing project to mitigate moral injury harms involves the design, implementation, and testing interventions that account for these barriers and facilitators elucidated by this research. Specifically, the need for peer support and organizational change highlighted by interviews informed the development of RECONN (Reflection and Connection), an organizational intervention designed by an interdisciplinary team to mitigate the impact of moral injury and to increase social support among nurses (37), built on these data combined with features of existing evidence based treatments for trauma and moral distress (22, 41, 52). Embedded within this quality improvement project is ongoing evaluation of its effectiveness and generalizability. This intervention is a promising improvement, but additional research is needed to support positive changes for nurses. Future manuscripts will detail the full development of RECONN and its initial implementation and scaling to additional units and hospitals. The overall implementation strategy and intervention results are/will be evaluated and reported utilizing Standards for Reporting Implementation Studies (StaRi) (52,53).

## Limitations

This research study has limitations, and findings should be interpreted with caution. First, the nurses belonged to a single healthcare system, on a single MICU. This potentially limits the generalizability to nurses working in other MICU outside of this institution as well as other ICUs within the organization. Additionally, this study included only one population—registered nurses. Including additional staff members, such as nurse managers and advanced practice providers (APPs, such as nurse practitioners) could expand our knowledge about barriers and facilitators of implementing an intervention on a MICU. These other nursing stakeholders have decision-making authority within the healthcare system and their insights can enhance our understanding of implementation decisions to integrate supportive interventions to mitigate moral injury. Finally, the composition of nurses' demographics is not racially diverse, is not representative of a varying age range, and does not represent the overall healthcare institution nurse demographics. This can contribute to constraints when considering expansion of interventions into other nursing units in this healthcare system and beyond.

## Conclusions

Implementation of interventions on nursing units face several challenges for widespread use; however, researchers can work with nurse stakeholders at various levels of a unit/institution to address what will and will not work on specific types of nursing units. To effectively address the pressures faced by nurses, high-level organizational change is needed, such as changes to structure, culture, operational methods, strategies, and technology. This way, the applicability and sustainability of generated interventions can be impactful to our nurses across healthcare settings.

## Data Availability

The data cannot be shared due to privacy concerns exacerbated by the small sample size and sensitive nature of the topic discussed. Data is available upon request with permission of the third party. Further queries can be directed to the corresponding author.
